# Different combinations of lignocellulolytic enzymes and lactic acid bacteria affect the quality and aerobic stability of rain-soaked Sudan grass silage by regulating the silage microbial community

**DOI:** 10.1128/spectrum.03547-25

**Published:** 2026-02-27

**Authors:** Mengxin Li, Qiang Yu, Ya Su, YiXi Long, Yuanjiang Rong, Hong Sun, Yixiao Xie, Jun Hao, Fuyu Yang, Yulong Zheng

**Affiliations:** 1 Guizhou University, College of Animal Science 71206, Guiyang, People's Republic of China; 2 China Agricultural University, College of Grassland Science & Technology 34752, Beijing, People's Republic of China; 3 China Agricultural University, Frontier Technology Research Institute, Shenzhen, People's Republic of China; University of Minnesota Twin Cities, St. Paul, Minnesota, USA

**Keywords:** Sudan grass silage, rainy, ferulic acid esterase, *Lactiplantibacillus plantarum*, degrading enzymes, aerobic stability, microbial community

## Abstract

This study evaluated the effects of ferulic acid esterase (FAE)-producing *Lactiplantibacillus plantarum* (LP; 1 × 10⁶ cfu·g⁻¹ FM), applied alone or with cellulase and xylanase (LCX; each enzyme 25 U·g⁻¹ FM) or cellulase and laccase (LCL; each enzyme 25 U·g⁻¹ FM), on the ensiling of rain-affected Sudan grass. After wilting to 75% moisture, the forage was vacuum-sealed in polyethylene bags and ensiled for 60 days, followed by 7 days of aerobic exposure. The LCX treatment significantly enhanced fermentation quality, yielding the lowest pH (3.83, *P <* 0.05), highest lactic acid (LA, 54.03 g·kg^−1^ DM, *P <* 0.05), and water-soluble carbohydrate (WSC, 21.45 g·kg^−1^ DM, *P <* 0.05) contents, and promoting the degradation of cellulose and hemicellulose after 60 days of ensiling. However, it resulted in the poorest aerobic stability. In contrast, the LCL treatment effectively reduced lignin and butyric acid (BA, 1.34 g·kg^−1^ DM, after 7 days of aerobic exposure, *P <* 0.05) contents, decreased fungal diversity, and increased acetic acid (AA, 4.82 g·kg^−1^ DM, *P <* 0.05), crude protein contents, and the relative abundance of *Lentilactobacillus* (*P <* 0.05), leading to superior aerobic stability exceeding 168 h, with stable nutritional and fermentation characteristics maintained after aerobic exposure. Both control and LP-only silages showed poor fermentation quality, with high pH (>5.16), substantial butyric acid production, nutrient loss, and proliferation of undesirable microbes such as *Enterobacter* and *Clostridium*. In conclusion, ensiling rain-soaked Sudan grass requires synergistic additives. While LCX enhances fermentation quality, LCL is recommended for effectively improving both fermentation quality and aerobic stability, offering a practical strategy for rainy regions.

**IMPORTANCE:**

Ensiling rain-soaked forages is a major challenge for livestock production in rainy regions, as it often leads to poor fermentation, nutrient loss, and rapid spoilage. This study demonstrates that tailored microbial-enzyme combinations can effectively overcome these issues. While adding specific enzymes with bacteria significantly improves the silage's nutritional value, it can make the silage prone to spoilage upon air exposure. Crucially, we found that incorporating a lignin-degrading enzyme (laccase) instead creates a more robust silage that resists spoilage for a remarkably longer time. This provides a practical and sustainable strategy for farmers to produce high-quality, stable silage from Sudan grass even when harvested under wet conditions, ensuring a reliable feed supply and supporting sustainable livestock farming in rainy climates like Southwest China.

## INTRODUCTION

The shortage of high-quality feed resources has become a major constraint on livestock production in China (1), making it vital to develop more high-quality feed resources. Sudan grass [*Sorghum sudanense* (Piper) Stapf], an annual forage grass belonging to the *Sorghum* genus and Gramineae family, is a C4 plant renowned as the “king of green feed” in aquaculture (2). It is characterized by high yield, strong stress resistance, rapid growth, and excellent regrowth ability; compared with corn and sorghum, Sudan grass exhibits other characteristics of softer stems and leaves, better palatability, and lower production costs, making it widely cultivated and used as animal feed (3–5). However, to improve the forage utilization efficiency of the grass, processing is often necessary. The production season of Sudan grass is in a rainy and humid climate of Southwest China; silage is often the only feasible preservation method (1, 6). Ensiling relies on lactic acid bacteria (LAB) fermentation to produce lactic acid (LA), which lowers the pH and inhibits harmful microorganisms of silage, thereby preserving the feed. Nevertheless, plant respiration, undesirable microbial growth, and clostridial fermentation can negatively impact the silages’ preservation during ensiling, leading to nutrient losses and reduced nutritional value (7). Sudan grass faces challenges, such as high fiber and moisture content, which impedes microbial activity, particularly after rainfall or under adverse weather conditions, resulting in suboptimal fermentation during silage production (8, 9).

In general, producing high-quality silage under unstable or rainy weather conditions remains challenging. The ensiling process requires appropriate moisture content and microorganisms of the raw material, as excessive moisture can promote undesirable clostridial fermentation (10, 11). Wilting is typically required to reduce moisture content, increase water-soluble carbohydrate (WSC) concentration, decrease water activity, and minimize effluent losses (12). However, in climatic conditions like those of southwestern China, forage often has high moisture content at harvest time and is difficult to reduce moisture. Delaying harvest to avoid the negative effects of the rain and high moisture is not cost-free, as it often leads to overmaturity and reduced nutritional value (13). Furthermore, rainfall significantly negatively affects forage ensiling by promoting direct leaching and increased plant respiration, leading to substantial losses of WSC; moreover, high humidity stimulates microbial and plant enzymatic activities, increasing the risk of mold growth and aerobic deterioration, which affects subsequent feed utilization (14, 15). Moreover, it is reasonable to assume that rainfall may alter the epiphytic microbial community structure on the raw material, further influencing fermentation quality (14).

Selecting suitable processing methods is critical to addressing the adverse effects of rainy weather on silage production and enhancing silage quality. Previous studies have shown that supplementing with exogenous LAB and providing more WSC after rainfall can improve the fermentation of silage (16). Furthermore, our previous research demonstrated that combining ferulic acid esterase (FAE)-producing *Lactiplantibacillus plantarum* with fibrolytic enzymes (xylanase and cellulase) significantly improved the silage quality, WSC content, and aerobic stability (9, 17). These results can be primarily attributed to the ability of feruloyl esterase to break the ester bonds between hemicellulose and lignin, which enhances the efficiency of fibrolytic enzymes, promoting the degradation of lignocellulose and the release of fermentable substrates, thereby improving LAB fermentation efficiency (18, 19). The combination of feruloyl esterase and fibrolytic enzymes also significantly increased the release of ferulic acid (6, 17, 18). The free ferulic acid is a phenolic compound with demonstrated antioxidant and antimicrobial activities (20). Additionally, the use of lignin-degrading enzymes (e.g., laccase and lignin peroxidase) could reduce lignin content and improve the digestibility of feed and also enhance the aerobic stability of corn and sugarcane silages (21). Xylanase primarily targets hemicellulose, which may more directly promote homolactic fermentation (22); laccase, as a multicopper oxidase, can catalyze the degradation of lignin and may produce phenolic oligomers or free radical intermediates with antibacterial activity (23). Moreover, another study of ours revealed that adding laccase in combination with FAE-producing *L. plantarum* not only enhanced silage quality but also significantly improved the aerobic stability of silage (24). However, for rain-affected forages, simultaneously improving both fermentation quality and aerobic stability is challenging. Additives that rapidly promote acidification can increase aerobic spoilage risk (25), while those focusing only on aerobic stability may hinder fermentation (26); hence, a balance is essential. Therefore, whether the combined application of degradative enzymes (cellulase, xylanase, and laccase) and FAE-producing *L. plantarum* can improve the quality and aerobic stability of rain-affected Sudan grass silage is of significant importance for the utilization of this forage in Southwest China.

Therefore, this study aims to explore the effects of adding FAE-producing *L. plantarum*, either alone or in combination with cellulase + xylanase, and cellulase + laccase, on the fermentation quality, aerobic stability, and microbial community structure of rain-affected Sudan grass silage. The findings are expected to provide theoretical support for the ensiling of Sudan grass in rain-prone regions during the harvest period.

## MATERIALS AND METHODS

### Test materials and additives

Whole-plant Sudan grass was obtained from the experimental station of Songtao Miao Autonomous County, Tongren City, Guizhou Province, located in Southwest China, 108°35′42″–109°23′30″ E and 27°49′40″−28°30′20″ N. The region has an annual average temperature of 16.5°C, a precipitation of 1,378.3 mm, and an average annual sunshine duration of 1,228 h. The Sudan grass was harvested at the heading stage (first cut) following a natural rainfall event of approximately 12 h with an accumulated precipitation of 20 mm, which occurred on the day of harvest in June 2023, with a stubble height of approximately 15 cm; the raw materials were immediately transported to the laboratory for silage preparation. The additives used in this experiment included FAE-producing *L. plantarum*, cellulase, xylanase, and laccase. The LAB was isolated and screened by our laboratory (6); cellulase and xylanase were sourced from Shanghai Macklin Biochemical Technology Co., Ltd.; laccase was obtained from Beijing Xiasheng Biotechnology Development Co., Ltd. For treatments involving enzymes (LCX and LCL), each enzyme was added at a rate of 25 U·g⁻¹ FM, resulting in a total enzyme activity of 50 U·g⁻¹ FM. The LP and CK treatments did not receive any enzymatic additives.

### Experimental design and silage preparation

The Sudan grass was chopped to 1–2 cm lengths via a guillotine and then wilted to approximately 75% moisture content. After thorough mixing, the material was randomly divided into four equal parts and subjected to the following treatments: (i) CK, addition of an equal amount of sterile distilled water; (ii) LP, adding FAE-producing *L. plantarum* alone with 1 × 10⁶ cfu·g⁻¹ fresh matter (FM); (iii) LCX, a combination of the LAB, cellulase, and xylanase; and (iv) LCL, a combination of LAB, cellulase, and laccase. The addition rate of each enzyme was 25 U·g⁻¹ FM, with a total amount of 50 U·g⁻¹ FM for all treatments (27). After thorough mixing, the materials were packed into polyethylene bags (28 × 35 cm) and vacuum-sealed. Each bag contained 300 g of material. A total of 100 bags (4 additive treatments × 5 ensiling days × 5 replications) were stored indoors (25°C ± 2°C) in the dark for 60 days, followed by aerobic exposure.

### Aerobic stability measurement

After 60 days of ensiling, the aerobic stability of the silage was tested according to the method described by Bai et al. (28). Briefly, silage bags were opened on day 60, and the material was loosened and transferred into sterile polyethylene buckets (5 L capacity; outer diameter: 28.5 cm, inner diameter: 24.6 cm, height: 18.5 cm). The buckets were covered with two layers of medical gauze to prevent cross-contamination and debris contamination. The buckets were kept in the dark at 27°C ± 1°C, and temperatures were recorded every 30 min using an automatic temperature recorder (TOPRIE TP9000, Shenzhen Toprie Electronics Co., Ltd.). Aerobic stability was defined as the time when the silage temperature exceeded the ambient temperature by 2°C (29). Nutritional characteristics, fermentation quality, and microbial communities were assessed after 0, 1, 3, 5, and 7 days of aerobic exposure.

### Fermentation characteristics and microbial population analysis

On the sampling day, 20 g of each sample was mixed with 180 mL of distilled water, then stored at 4°C for 24 h. The mixture was filtered through four layers of gauze, and the filtrate was divided into four portions. One portion was immediately used for pH measurement with a pH meter (PHSJ-5T, INESA Scientific Instrument Co., Ltd., Shanghai, China). Another portion was filtered through a 0.22 μm membrane and analyzed for lactic acid (LA), acetic acid (AA), propionic acid (PA), and butyric acid (BA) using high-performance liquid chromatography (HPLC, Thermo Fisher Scientific, USA) following the method described by Li et al. (30) (column: KC-811; column temperature: 50°C; flow rate: 1 mL min⁻¹). Ammonia nitrogen (AN) content was determined according to the method described by Broderick and Kang (31).

Microbial counts were analyzed by the plate count method. Serial dilutions (10⁻¹–10⁻⁷) of each sample extract (100 µL) were prepared using sterile distilled water. Aliquots (10 µL) from the 10⁻³, 10⁻⁵, and 10⁻⁷ dilutions were spread on agar plates. LAB were counted on MRS (De Man, Rogosa, and Sharpe) agar plates after incubation at 37°C under anaerobic conditions for 48 h; yeasts/molds and coliforms were counted on Bengal red agar and eosin methylene blue agar plates, respectively, after incubation at 30°C for 48 h. All culture media were obtained from Beijing Luqiao Technology Co., Ltd.

Fresh raw and silage samples were dried at 65°C for 72 h until constant weight to determine dry matter (DM) content. The dried samples were ground using a mill (DLF-18B, Wenzhou Top Medical Instrument Co., Ltd., Zhejiang, China) and then passed through a 40-mesh sieve for chemical analysis. The contents of neutral detergent fiber (aNDF), acid detergent fiber (ADF), and acid detergent lignin (ADL) were determined using a fiber analyzer (ANKOMDELTA, ANKOM Technology, Macedon, NY, USA) according to Van Soest et al. (32) with a heat-stable amylase (FAA, Ankom Technology, Macedon, NY), and the analysis included measurement of the residual acid-insoluble ash (AIA). Crude protein (CP) content was determined by the Kjeldahl method (VAPODEST500, C. Gerhardt GmbH & Co. KG, Germany). Water-soluble carbohydrate (WSC) content was measured following the method described by Murphy (33).

### Microbial community analysis

An additional 10 g of each sample was stored in sealed bags at −80°C for microbial community analysis. To further elucidate microbial dynamics of fresh material and samples subjected to 0, 3, and 7 days of aerobic exposure, DNA was extracted using the standard cetyltrimethylammonium bromide (CTAB) method for sequencing and strain identification. Polymerase chain reaction (PCR) amplification was performed using barcoded primers and GC buffer from New England Biolabs (Ipswich, MA, USA), along with high-fidelity and high-efficiency enzymes. Bacterial 16S rRNA genes were amplified with primers F (5′-CCTAYGGGRBGCASCAG-3′) and R (5′-GGACTACNNGGGTATCTAAT-3′). Fungal ITS rRNA genes were amplified with primers F (5′-CTTGGTCATTTAGAGGAAGTAA-3′) and R (5′-GCTGC GTTCTTCATCGATGC-3′). The amplification protocol was as follows: initial denaturation at 95°C for 2 min; 30 cycles of denaturation at 95°C for 30 s, annealing at 55°C for 30 s, and extension at 72°C for 30 s; final extension was at 72°C for 5 min. A small-fragment library was constructed based on the amplified regions. Species composition and abundance analysis were conducted by Novogene Technology Co., Ltd (Beijing, China). Alpha diversity and coverage values were analyzed using the Magic platform (https://magic.novogene.com/) to evaluate community structure differences.

### Statistical analysis

The hemicellulose, cellulose, and lignin contents were estimated using equations 1-3, respectively.


(1) $$Hemicellulose\;Content\;(g\cdot kg^{-1})=NDF\;Content\;(g\cdot kg^{-1})-ADF\;Content\;(g\cdot kg^{-1})$$

(2) $$Cellulose\;Content\;(g\cdot kg^{-1})=ADF\;Content\;(g\;kg^{-1})-ADL\;Content\;(g\;kg^{-1})$$

(3) $$Lignin\;Content\cdot (g\cdot kg^{-1})=ADL\;Content\;(g\cdot kg^{-1})-AIA\;Content\;(g\cdot kg^{-1})$$

The fixed effects of additives and aerobic exposure time were analyzed using multifactorial analysis of variance (one-way or two-way) in SPSS 27 software (IBM Corp., New York, NY, USA). Microbial count data were normalized by log₁₀ transformation. Statistical differences between means were determined using Duncan’s multiple comparison test. Differences were considered significant at *P* < 0.05.

## RESULTS AND DISCUSSION

### Chemical composition of raw materials before ensiling

The chemical composition and microbial counts of the raw Sudan grass material are presented in Tables 1 to 3. The DM content of the material was 239.42 g kg⁻¹ FM, which was achieved through wilting. The CP content was 82.51 g kg⁻¹ DM, consistent with the findings reported by Guo et al. (1) but lower than that reported by Wan et al. (34). Such variations may be attributed to differences in climatic conditions, soil fertility, harvesting time, and irrigation practices (35). WSC and epiphytic LAB are critical factors for ensuring well-preserved silage. Generally, WSC content greater than 60 g·kg⁻¹ DM is required to adequately support LAB growth and fermentation (9, 30). In this study, the WSC content of raw materials was 45.56 g kg⁻¹ DM, significantly lower than that observed in the second cut of Sudan grass from the same experimental field (9). This difference may be due to variations in harvesting time or losses caused by rainfall (14; 15). Low WSC content may promote the proliferation of undesirable bacteria such as clostridia, leading to the production of detrimental metabolites and nutritional losses during ensiling (36). A high-quality silage typically requires an epiphytic LAB count of at least 5 lg cfu·g⁻¹ FM (37). The epiphytic LAB count in the material was 5.81 log cfug⁻¹ FM, indicating that effective fermentation can be conducted under sufficient WSC conditions. However, the coliform count reached 6.21 log cfug⁻¹ FM, and the increased undesirable microorganisms were likely due to the rainfall. These microbes may compete with LAB and negatively impact silage quality. Additionally, the NDF, ADF, and ADL contents were 657.2, 389.5, and 40.32 g kg⁻¹ DM, respectively. High lignocellulose content may adversely affect animal intake and digestibility.

**TABLE 1:** Characteristics of fresh material of Sudan grass (*n* = 3)^*a*^

Item	Mean ± standard deviation
pH	6.06 ± 0.08
Dry matter (g·kg^−1^ FM)	239.42 ± 1.17
Water soluble carbohydrates (g·kg^−1^ DM)	45.56 ± 1.22
Crude protein (g·kg^−1^ DM)	82.51 ± 7.81
Neutral detergent fiber (g·kg^−1^ DM)	657.2 ± 6.11
Acid detergent fiber (g·kg^−1^ DM)	389.5 ± 4.31
Cellulose (g·kg^−1^ DM)	344.9 ± 4.11
Hemicellulose (g·kg^−1^ DM)	267.7 ± 1.99
Acid detergent lignin (g·kg^−1^ DM)	40.32 ± 1.21
Acid insoluble ash (g·kg^−1^ DM)	4.19 ± 0.20
Lactic acid bacteria (log_10_ cfu·g^−1^ FM)	5.81 ± 0.07
Yeast (log_10_ cfu·g^−1^ FM)	4.69 ± 0.01
Coliform (log_10_ cfu·g^−1^ FM)	6.21 ± 0.02

^*a*^ FM, fresh matter; DM, dry matter; cfu, colony-forming unit.

**TABLE 2:** Dynamic changes of fermentation parameters during aerobic exposure of Sudan grass silage^*a*^

Item	Treatment	Ensiling time	Aerobic exposure time				Mean	SEM	*P*-value		
		Day 60	Day 1	Day 3	Day 5	Day 7			T	D	T × D
LA (g·kg^−1^ DM)	CK	3.78 Da	3.51 Dab	3.29 Cb	1.94 BCc	0.68 Bd	2.64	2.063	<0.001	<0.001	<0.001
	LP	5.82 Ca	5.56 Ca	4.57 Cb	3.04 Bc	1.30 Bd	4.06				
	LCX	54.03 Aa	50.49 Ab	46.91 Ac	1.31 Cd	0.42 Bd	30.63				
	LCL	45.34 Ba	44.14 Ba	43.49 Ba	35.14 Ab	28.14 Ac	39.25				
AA (g·kg^−1^ DM)	CK	1.76 C	1.82 C	1.82 B	1.79 B	1.84 A	1.81	0.148	<0.001	<0.001	<0.001
	LP	1.19 Db	1.58 Ca	1.71 Ba	1.59 Ba	1.69 Aa	1.55				
	LCX	2.59 Ba	2.34 Bb	1.16 Cc	0.20 Cd	0.18 Cd	1.29				
	LCL	4.82 Aa	4.96 Aa	4.95 Aa	4.71 Aa	1.06 Bb	4.10				
LA/AA	CK	2.23 Da	2.06 Da	1.84 Ca	1.09 Bb	0.38 Cc	1.52	1.056	<0.001	<0.001	<0.001
	LP	5.12 Ca	3.67 Cb	2.71 Cbc	1.91 Bc	0.78 Cd	2.84				
	LCX	20.89 Ab	21.65 Ab	41.06 Aa	6.52 Ac	2.36 Bd	18.49				
	LCL	9.51 Bb	8.92 Bb	8.79 Bbc	7.47 Ac	26.58 Aa	12.26				
PA (g·kg^−1^ DM)	CK	0.00	0.00	0.00	0.00	0.00	0.00	0.008	<0.001	<0.001	<0.001
	LP	0.00	0.00	0.00	0.00	0.00	0.00				
	LCX	0.00	0.00	0.00	0.00	0.00	0.00				
	LCL	0.05 C	0.09 BC	0.12 BC	0.15 B	0.29 A	0.14				
BA (g·kg^−1^ DM)	CK	11.58 C	14.05 AB	14.09 ABa	13.35 Ba	15.56 Aa	13.73	0.656	<0.001	<0.001	<0.001
	LP	11.33 CD	13.26 B	12.23 BCb	10.48 Db	15.18 Aa	12.49				
	LCX	0.00	0.00	0.00	0.00	2.89 b	0.58				
	LCL	0.00	0.00	0.00	0.00	1.34 c	0.27				
AN (g kg-1 TN)	CK	43.31 Ac	44.21 Ac	44.37 Ac	49.83 Ab	57.59 Aa	47.86	1.564	<0.001	<0.001	<0.001
	LP	38.29 Bc	38.18 Bc	40.45 Bc	45.21 Bb	50.77 Ba	42.59				
	LCX	18.15 Db	19.76 Da	18.11 Db	3.78 Dc	4.05 Dc	12.77				
	LCL	21.49 Cab	22.61 Cab	23.24 Ca	21.00 Cb	16.71 Cc	21.01				
Lactic acid bacteria (log_10_ cfu g^−1^ FM)	CK	7.45 Cc	7.47 Cc	7.57 Bc	7.60 Bc	7.77 Ab	7.57	0.232	<0.001	<0.001	<0.001
	LP	7.69 Bb	7.67 Bb	7.69 Bb	7.73 Bb	7.94 Aa	7.74				
	LCX	ND	ND	7.09 Bd	7.05 Bd	7.24 Ac	4.28				
	LCL	7.95 BCa	7.94 BCa	7.92 Ca	7.96 Ba	8.01 Aa	7.96				
Yeast (log_10_ cfu g^−1^ FM)	CK	ND	ND	ND	ND	7.29 c	1.46	0.364	<0.001	<0.001	<0.001
	LP	ND	ND	ND	ND	7.09 d	1.42				
	LCX	ND	ND	7.45 C	8.45 B	9.02 Aa	4.98				
	LCL	ND	ND	ND	ND	7.92 b	1.58				
Coliform (log_10_ cfu g^−1^ FM)	CK	7.29 Ca	7.32 Ca	7.72 Ba	7.73 Ba	7.86 Ab	7.59	0.331	<0.001	<0.001	<0.001
	LP	7.14 Db	7.22 Cb	7.59 Bb	7.63 Bb	7.84 Ab	7.49				
	LCX	ND	ND	7.06 Cc	7.72 Ba	8.56 Aa	4.67				
	LCL	ND	ND	ND	7.04 Bc	7.67 Ac	2.94				
Mold (log_10_ cfu g^−1^ FM)	CK	ND	ND	ND	ND	ND	–	–	–	–	–
	LP	ND	ND	ND	ND	ND	–				
	LCX	ND	ND	ND	ND	ND	–				
	LCL	ND	ND	ND	ND	ND	–				

^*a*^ LP,* L. plantarum*; LCX, *L. plantarum* + cellulase + xylanase; LCL,* L. plantarum* + cellulase + laccase; ND, not detected; –, not applicable; SEM, standard error of the mean; D, ensiling day; T, additive; T*D, interaction between ensiling day and additive. Different lowercase letters indicate significant differences among different treatments at the same time (*P *< 0.05). Different uppercase letters indicate significant differences among different times within the same treatment (*P *< 0.05).

**TABLE 3:** Alpha diversity of bacteria and fungi in Sudan grass silage on 60 days of ensiling and during aerobic exposure^*a*^

Days	Treatment	Bacteria				Fungi			
		Chao1	Shannon	OTUs	Good’s Coverage	Chao1	Shannon	OTUs	Good’s Coverage
	Fresh	797.22 b	7.69 b	794.67 b	0.9989 ab	322.24 ab	4.06 ab	303.67 ab	0.9993 b
60 d	CK	198.90 de	5.01 c	197 de	0.9995 a	209.69 bc	4.73 ab	207 bc	0.9999 a
	LP	126.88 e	4.49 c	126 e	0.9996 a	283.81 ab	5.13 a	280 ab	0.9999 a
	LCX	100.84 e	2.91 d	97 e	0.9993 ab	249.08 b	5.79 a	247 b	0.9999 a
	LCL	75.07 e	2.92 d	70 e	0.9965 bc	225.71 bc	4.31 ab	217 bc	0.9997 a
Day 3	CK	208.50 de	5.28 c	205 de	0.9991 ab	361.20 a	5.24 a	356 a	0.9998 a
	LP	116.46 e	4.59 c	116 e	0.9998 a	249.88 b	4.98 b	244 b	0.9997 a
	LCX	473.29 c	4.62 c	459 c	0.9998 a	41.23 de	1.45 cd	37 de	0.9999 a
	LCL	472.81 c	4.47 c	454 c	0.9956 c	227.05 bc	4.40 ab	215 bc	0.9997 a
Day 7	CK	181.76 de	4.67 c	177 de	0.9991 ab	39.99 de	0.60 d	44 de	0.9999 a
	LP	142.55 e	5.02 c	136 e	0.9986 ab	128.63 cd	2.93 bc	127 cd	0.9999 a
	LCX	411.85 cd	5.01 c	401 cd	0.9978 abc	24.17 de	1.93 cd	24 de	0.9999 a
	LCL	1458.89 a	8.99 a	1428 a	0.9930 d	11.78 e	1.50 cd	12 e	0.9999 a
SEM		63.45	0.27	62.20	0.0004	20.28	0.30	19.74	0.00003
*P*-value		<0.001	<0.001	<0.001	<0.001	<0.001	<0.001	<0.001	<0.01

^*a*^ LP,* L. plantarum*; LCX, *L. plantarum* + cellulase + xylanase; LCL,* L. plantarum* + cellulase + laccase; SEM, standard error of the mean; 60 d, 60 days of ensiling; Day 3, aerobic exposure of 3 days; Day 7, aerobic exposure of 7 days. Different lowercase letters indicate significant differences among different treatments within the same time (*P *< 0.05).

### Aerobic stability and pH changes of Sudan grass silage

**Fig 1:**
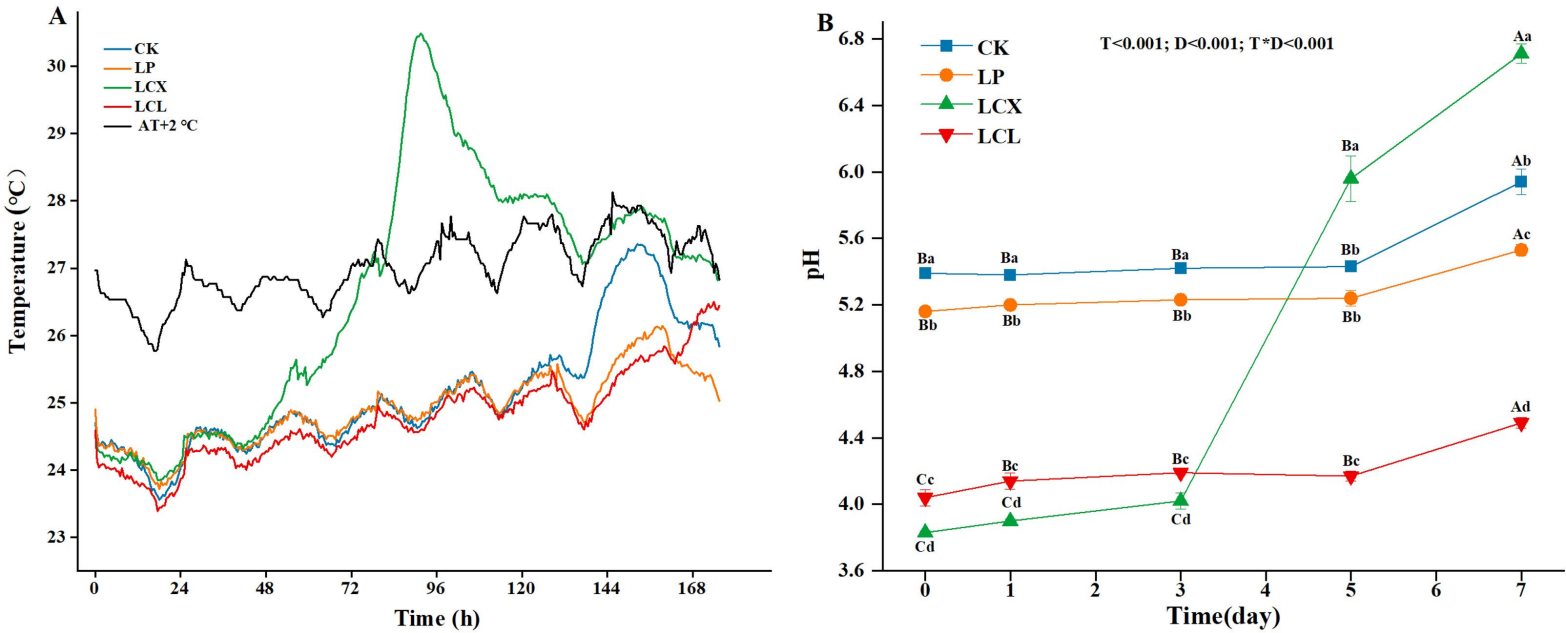
Effect of different treatments on the temperature (**A**) and pH (**B**) dynamics of Sudan grass silage during the aerobic exposure. LP, *L. plantarum*; LCX, *L. plantarum* + cellulase + xylanase; LCL, *L. plantarum* + cellulase + laccase; AT, ambient temperature + 2°C. D, ensiling day; T, additive; T*D, interaction between ensiling day and additive.

High-quality silage is often susceptible to aerobic deterioration after opening due to the presence of residual WSC and high LA content, which serve as metabolic substrates for aerobic microorganisms such as yeasts. This microbial activity leads to the production of CO₂ and water, releasing excessive heat and causing a rise in temperature, ultimately resulting in silage spoilage (38). Therefore, temperature is a key indicator for evaluating silage spoilage. Generally, the onset of spoilage is defined as the time when the internal temperature of the silage exceeds the ambient temperature by 2°C (38). The temperature changes during aerobic exposure of Sudan grass silage are shown in Fig. 1A. In this study, the LCX treatment (LAB + cellulase + xylanase) was the first to exhibit aerobic spoilage, with its temperature exceeding the ambient temperature by 2°C at about 79 h. This observation is consistent with previous findings that silage treated with LAB and exogenous enzymes is prone to aerobic deterioration first (39). The reason may be attributed to the synergistic effect of the bacterial-enzyme combination on lignocellulose degradation, which generated more fermentation substrates, resulted in higher LA production and more residual WSC compared to other treatments (Table 2; Fig. 3C). Well-fermented silage with a high LA content tends to be unstable during aerobic exposure (40). When exposed to air, LA can be utilized by yeasts and other aerobic microorganisms, leading to a rise in pH and promoting the growth of acid-intolerant undesirable microbes (41). While both CK and LP (adding LAB alone) treatments did not show early deterioration, with more than 144 h of aerobic stability, this may be due to their higher BA content (Table 2), as BA inhibits the growth of fungi such as yeasts, thereby improving aerobic stability (42). Danner et al. (43) previously reported that silage with a BA content exceeding 5 g kg⁻¹ DM exhibits significantly enhanced aerobic stability. In terms of temperature dynamics, the LP demonstrated better aerobic stability than CK, likely because the addition of FAE-producing *L. plantarum* increased the free ferulic acid content during ensiling, which possesses antioxidant and antimicrobial properties (17, 20). Notably, the LCL treatment showed no signs of aerobic deterioration, maintaining aerobic stability for over 168 h. This is possibly due to the phenolic substances (such as ferulic acid, p-coumaric acid, etc.) released by peroxidase during the degradation of lignin, which have broad-spectrum antibacterial activity against aerobic spoilage microorganisms such as yeasts and molds (24, 44), as well as the higher AA content and the presence of PA (Table 2). Both AA and PA are effective antifungal agents and are recognized as efficient inhibitors of aerobic deterioration in silage (29, 43).

**Fig 2:**
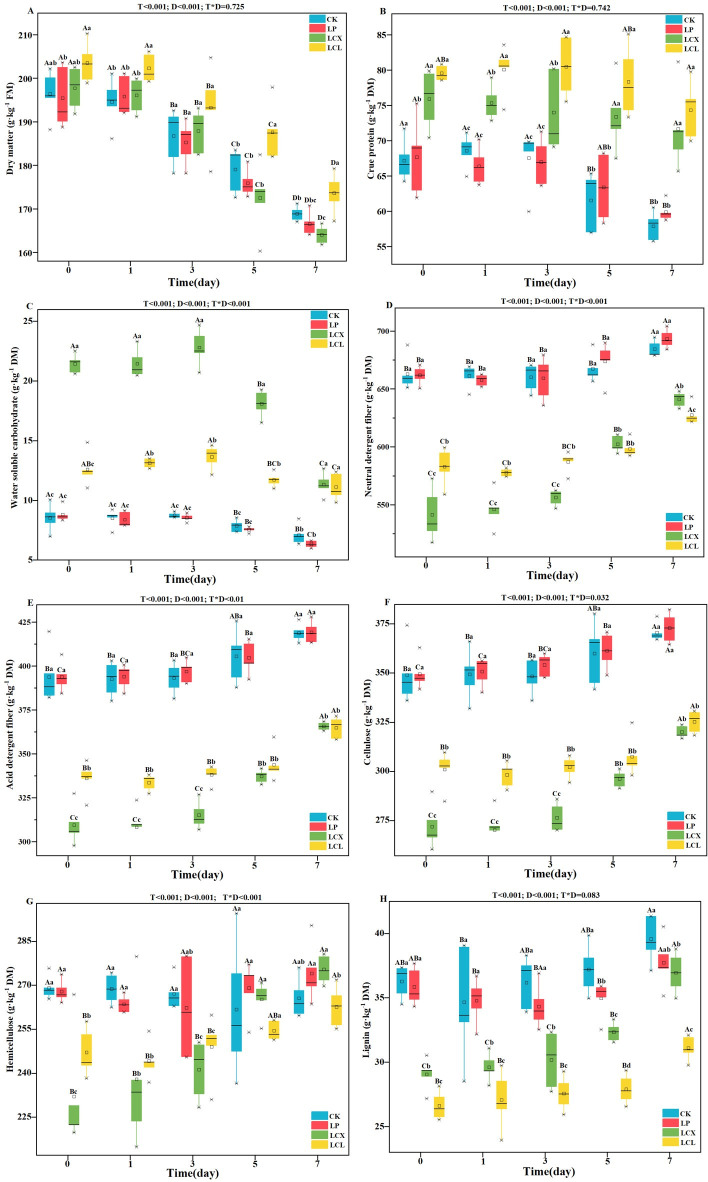
Dry matter (**A**), crude protein (**B**), water-soluble carbohydrate (**C**), neutral detergent fiber (**D**), acid detergent fiber (**E**), cellulose (**F**), hemicellulose (**G**), and lignin (**H**) content of Sudan grass silage during aerobic exposure. LP, *L. plantarum*; LCX, *L. plantarum* + cellulase + xylanase; LCL, *L. plantarum* + cellulase + laccase; D, ensiling day; T, additive; T*D, interaction between ensiling day and additive.

High-quality silage should exhibit a pH below 4.2 (12), with LA serving as the primary organic acid responsible for pH reduction. As shown in Fig. 1B, all additive treatments significantly lowered pH after 60 days of ensiling compared to the CK group (*P* < 0.05). However, only the combined bacterial-enzyme treatments (LCX and LCL) achieved a pH below 4.2, indicating well-preserved silage. This aligns with findings by Mu et al. (39), likely due to the synergistic action of the enzymes and bacteria promoting lignocellulose decomposition and increasing fermentable substrates for LAB metabolism. While our previous studies showed that adding FAE-producing LAB alone could reduce the pH of Sudan grass silage to approximately 4.0, the LP group in this study only reached a pH of around 5.0. This discrepancy may be attributed to rainfall, which induced reductions in WSC content and higher levels of undesirable epiphytic microorganisms (14; 15). Compared to LCL, LCX demonstrated superior fermentation performance (with a lower pH); this can be explained by the different substrates targeted by xylanase and laccase. Xylanase specifically releases more WSC (Fig. 2C), thereby providing more fermentable sugars for LAB. The epiphytic LAB count in the raw material exceeded 10⁵ CFU/g FM, which is considered sufficient to initiate effective silage fermentation (45), but the CK and LP exhibited relatively high pH values. This outcome may be attributed to rainfall prior to ensiling, which likely resulted in a high coliform count (6.21 log CFU/g FM) and reduced WSC content in the raw material (Table 1), thereby impairing the competitiveness of LAB. These results further emphasize that FAE-producing *L. plantarum* requires synergistic interaction with fibrolytic enzymes to effectively degrade lignocellulose and enhance LA fermentation. During aerobic exposure, all treatments showed a significant pH increase by day 7 (*P* < 0.05). This phenomenon results from the rapid proliferation of aerobic microorganisms (e.g., lactate-assimilating yeasts) upon air infiltration, which metabolize LA and raise pH, subsequently promoting the growth of acid-intolerant microbes and accelerating spoilage (39, 46). Thus, pH can serve as a key indicator of aerobic stability. According to Yuan et al. (46), aerobic stability is defined as the time before pH increases by more than 0.5 units from the initial value. Consistent with temperature trends, only CK and LCX exceeded this threshold, with LCX being the first to surpass the critical value on day 5.

### Fermentation characteristics and microbial counts of Sudan grass silage

**Fig 3:**
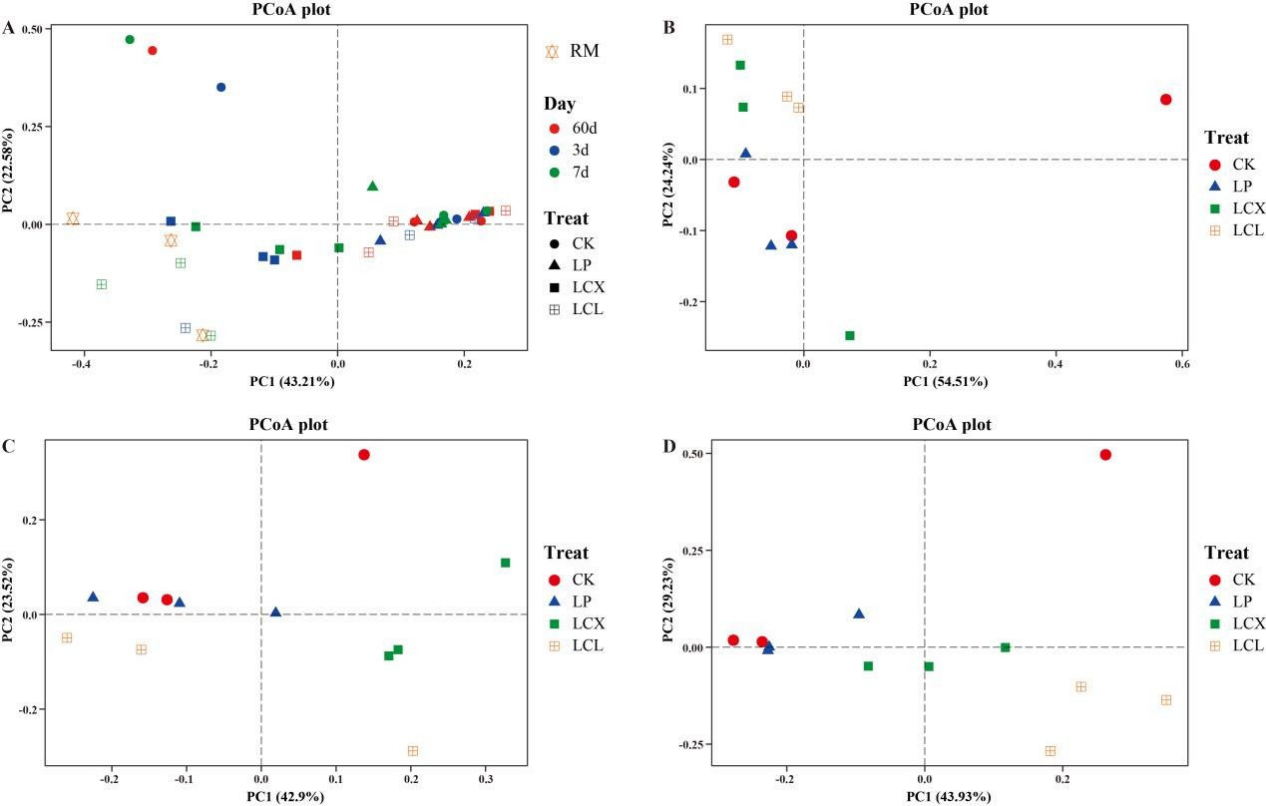
Principal coordinate analysis of bacterial communities. (**A**) All time periods; (**B**) 60 days of ensiling; (**C**) aerobic exposure of 3 days; and (**D**) aerobic exposure of 7 days. LP, *L. plantarum*; LCX, *L. plantarum* + cellulase + xylanase; LCL, *L. plantarum* + cellulase + laccase.

**Fig 4:**
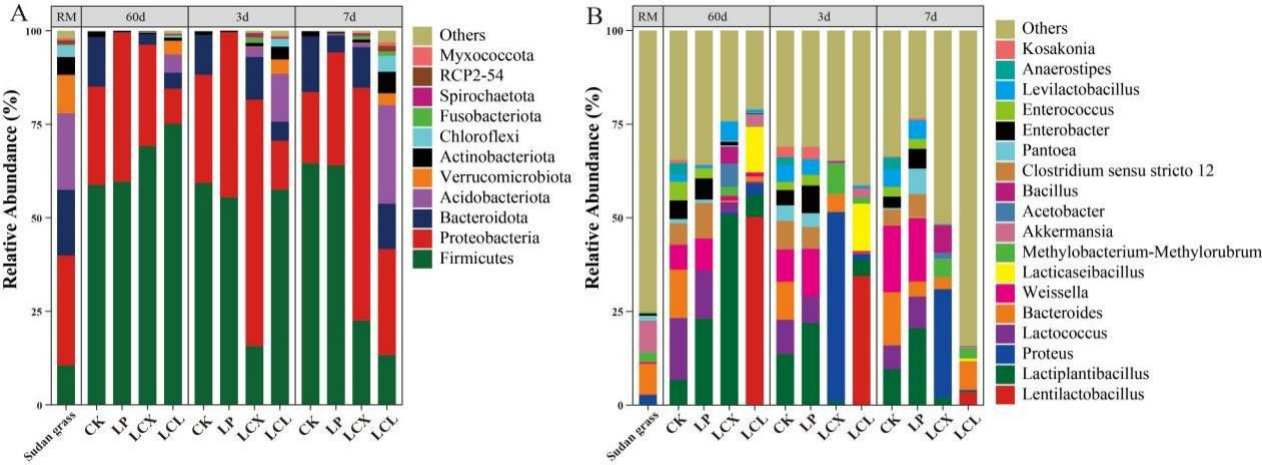
Composition of bacterial communities on 60 days of ensiling and during aerobic exposure of Sudan grass. (**A**) Phylum level; (**B**) genus level. LP, *L. plantarum*; LCX, *L. plantarum* + cellulase + xylanase; LCL, *L. plantarum* + cellulase + laccase.

The changes in fermentation characteristics of Sudan grass silage after 60 days of ensiling and during the aerobic exposure are shown in Fig. 1 and Table 2. The interaction between additives and aerobic exposure time had a significant effect on the contents of LA, AA, LA/AA ratio, PA, BA, and AN (*P* < 0.001). Compared to the CK, all additive treatments significantly increased the LA content after 60 days of ensiling (*P* < 0.05). However, the lower LA content in CK and LP was likely due to competition from a higher coliform population and lower WSC content. Furthermore, the LA content in all treatments decreased significantly by day 5 of aerobic exposure (*P* < 0.05), primarily due to consumption by aerobic microorganisms. However, for the later stage of aerobic exposure and average values of each treatment, the LCL treatment group maintained a significantly higher LA content than other groups (*P* < 0.05). This indicates that aerobic microbial activity was suppressed, leading to better preservation of LA, further corroborating the superior aerobic stability of the LCL treatment. Moreover, the LCL treatment resulted in a significantly higher AA content after 60 days compared to other treatments (*P* < 0.05), and this was mainly attributed to the higher relative abundance of the genus *Lentilactobacillus* in the LCL treatment (Fig. 3B). *Lentilactobacillus* is a heterofermentative LAB that can produce LA and AA (10, 47). Therefore, it significantly improved the aerobic stability of the LCL group (Fig. 1A). Usually, the AA in the silage is metabolized and utilized by aerobic microorganisms upon air exposure. However, the AA content in CK and LP increased during the aerobic exposure period. This might be due to the higher relative abundance of *Weissella* species in CK and LP (Fig. 4B), which belong to heterofermentative LAB and are relatively sensitive to low pH (45).

Except for CK, all treatments exhibited an LA/AA ratio greater than 3, indicating a predominantly homolactic fermentation pattern. The LCX treatment showed the highest ratio (*P* < 0.05), which can be attributed to the addition of homofermentative FAE-producing *L. plantarum* and the greater availability of fermentable substrates produced by bacteria-enzyme treatment. A small amount of PA, which also possesses antimicrobial properties, was detected only in the LCL treatment. This finding partially explains the higher aerobic stability observed in the LCL group. After 60 days of ensiling, high BA content was detected only in the CK and LP treatments, indicating poor fermentation in these groups and suggesting that using no additives or adding only FAE-producing *L. plantarum* is ineffective for ensiling rain-affected Sudan grass. Because this organic acid is generally an undesirable end product in silage, which should be less than 10 g kg⁻¹ DM in high-quality silage, as its presence can reduce feed intake and increase the risk of ketosis in animals (36). However, BA has demonstrated antifungal properties and can inhibit aerobic microbes (48), and this might be one of the reasons why CK has high aerobic stability. The AN content in silage is associated with the degradation of CP. Compared to the CK, all additive treatments (LP, LCX, and LCL) significantly reduced the AN content after ensiling for 60 days (*P* < 0.05), primarily because their lower pH values inhibited the activity of plant enzymes and proteolytic bacteria (49). After 5 days of aerobic exposure, the AN content increased significantly in CK and LP (*P* < 0.05), indicating the onset of spoilage and intensified CP degradation by undesirable microorganisms. Interestingly, the AN content decreased significantly by day 7 of aerobic exposure in the LCX and LCL treatments (*P* < 0.05), a phenomenon that requires further investigation.

The changes in microbial populations of Sudan grass silage are presented in Table 2. The interaction between additives and aerobic exposure time significantly affected the counts of LAB, coliform, and yeast (*P* < 0.001). The essence of silage fermentation is a process driven by LAB. As expected, the LP and LCL treatments increased the LAB count after 60 days of ensiling, with the LCL treatment showing a higher LAB count than other treatments (*P* < 0.05). This indicates a synergistic effect between the added LAB, cellulase, and laccase. Interestingly, no LAB were detected in the LCX treatment, and this could be due to the relatively low pH (3.83) in this treatment, as an excessively low pH environment can also inhibit LAB growth (50). Therefore, we can only use the plate counting method as a reference because the plate counting method may underestimate the number of culturable bacteria in extremely acidic environments, while microbial community analysis can provide a more comprehensive reflection of the existence of the community. During the aerobic exposure, the LAB counts did not decrease in any treatment; instead, they increased significantly by day 7 (*P* < 0.05). This result is consistent with the findings of Zheng et al. (51) and can be attributed to the rise in pH after aerobic exposure, which allowed the growth of LAB species (such as *Weissella*, *Lactococcus,* and *Enterococcus* in Fig. 4B) that are intolerant to low pH (45). After 60 days of ensiling, no yeasts or molds were detected in any treatment, while coliform was only detected in the CK and LP treatments. This is likely because the anaerobic conditions in the later stages of fermentation inhibited yeast growth, and the lower pH in the LCX and LCL treatments suppressed coliform activity (52). Molds were only detected in the CK treatment during the later stage of aerobic exposure, and the counts of yeasts and coliforms increased with the duration of aerobic exposure. This is similar to the findings of Zheng et al. (51), which can be attributed to the fact that it was an aerobic environment and the increase in pH.

### Nutritional and fiber composition of Sudan grass silage

As presented in Fig. 2, the interaction between additives and aerobic exposure time significantly affected WSC, NDF, and hemicellulose content (*P* < 0.001), cellulose content (*P* < 0.01), and ADF content (*P* < 0.05). Compared to other treatments, the LCL treatment effectively preserved DM content (*P* < 0.05). This is likely attributable to its higher AA content and superior aerobic stability, which effectively suppressed the activity and growth of undesirable microorganisms and aerobic spoilage microbes. After 60 days of ensiling, both the LCX and LCL treatments effectively preserved the CP content and significantly increased WSC content compared to the CK and LP treatments (*P* < 0.05). This can be attributed to their lower pH values inhibiting plant proteases and the activity of proteolytic undesirable microorganisms (49), coupled with the synergistic degradation of lignocellulose by the FAE-producing *L. plantarum* and degrading enzyme, which released more WSC (18, 53). During the aerobic exposure, the DM, CP, and WSC contents decreased significantly in all treatments (*P* < 0.05). This trend aligns with the observed changes in temperature and pH and is primarily due to the utilization of organic components like CP and WSC by aerobic microorganisms, leading to DM losses.

The levels of the fiber components could affect animal feed intake. In this study, after 60 days, the LCX and LCL treatments significantly reduced the contents of NDF, ADF, cellulose, and hemicellulose compared to the CK and LP treatments (*P* < 0.05), which could promote the animal utilization of Sudan grass forage. This result aligns with findings by Li et al. (18) and is likely due to the synergistic effect of combining FAE-producing *L. plantarum* with cellulase, xylanase, and laccase (9, 17). The mechanism can be explained by considering the inherent structure of lignocellulose and the synergistic degradation pathways facilitated by the additives: plant lignocellulose is primarily composed of cellulose (38%–50%), hemicellulose (23%–32%), and lignin (10%–25%) (54), within which hemicellulose and lignin form a complex via ether and ester bonds that surround cellulose and restrict cellulase accessibility (55). The bacterial-enzyme synergy disrupts this barrier through multiple pathways: the feruloyl esterase produced by the LAB addition breaks the lignin-hemicellulose ester bonds, compromising cell wall integrity, thereby enhancing the access of exogenous enzymes and acids to polysaccharides (30, 56); meanwhile, the acids produced during fermentation enhance the hydrolysis process. As the acidity increases, the lignocellulose will be further degraded (57). Due to their specific enzymatic properties, the exogenous enzymes (cellulase, xylanase, and laccase) target and degrade their respective components. Therefore, the LCX group (containing xylanase and cellulase) performed better in the degradation of cellulose and hemicellulose, while the LCL group (containing laccase) excelled in the removal of lignin. Additionally, hemicellulose, with its amorphous structure and lower degree of polymerization compared to cellulose, is highly susceptible to hydrolysis (58). This explains the more effective degradation of cellulose and hemicellulose in the LCX treatment, as also evidenced by its higher WSC content. Lignin is an amorphous, highly branched aromatic polymer primarily located in the plant secondary cell wall and is notoriously difficult to degrade during ensiling (59). In this study, the LCL treatment exhibited the lowest lignin content (*P* < 0.05), attributable to the strong degradative capability of laccase against lignin (59), and synergistic effect with FAE-producing *L. plantarum* and cellulase. The contents of NDF, ADF, cellulose, hemicellulose, and lignin increased with the duration of aerobic exposure in all treatments (*P* < 0.05), which can be attributed to the substantial proliferation of aerobic bacteria during aerobic exposure; substances like soluble sugars and proteins will be metabolized and converted into CO_2_, resulting in their loss. Consequently, the relative proportion of the more resistant fiber components (cellulose, hemicellulose, and lignin) increases correspondingly (9, 27).

### Bacterial community of Sudan grass silage during aerobic exposure

The bacterial alpha diversity of Sudan grass silage is presented in Table 3. The Good’s coverage values for all treatments exceeded 0.99, indicating that the sequencing depth sufficiently captured the majority of the bacterial communities (6). The Chao1 index reflects species richness within the samples, while the Shannon index represents species diversity. After 60 days of ensiling, the LP treatments (LP, LCX, and LCL) showed reduced bacterial richness and diversity compared to the CK group, especially in the LCL treatment. Similarly, previous studies have reported that inoculating LAB can lead to a decrease in bacterial diversity of silage, often due to an increase in the relative abundance of a dominant genus (e.g., *Lactobacillus*) and the suppression of undesirable bacterial growth under lower pH conditions (60). The lowest bacterial diversity in the LCL treatment might be further attributed to its higher AA content, which effectively inhibited the growth of undesirable bacteria. After 7 days of aerobic exposure, bacterial richness and diversity increased in all treatments. The reason might be the rise in pH after aerobic exposure, creating favorable conditions for the proliferation of various spoilage microorganisms that are less acid-tolerant (61).

As shown in Fig. 3, the fresh Sudan grass samples were primarily clustered in the third quadrant, whereas the ensiled samples were mainly distributed in the first and fourth quadrants, showing significant separation between the two groups (Fig. 3A). This finding is consistent with the results reported by Ni et al. (49). The observed separation can be largely attributed to the suppression of many epiphytic bacteria in the fresh material under anaerobic fermentation conditions. After 60 days of ensiling, the CK and LP treatments were predominantly distributed in the third quadrant, while the LCX and LCL treatments were mainly situated in the second quadrant, indicating that the combined addition of bacteria and enzymes modulated the bacterial community structure. Spoilage was observed in the LCX treatment after 3 days of aerobic exposure, which likely explains its distinct separation from the other treatment samples (Fig. 3C and D). The position of the LCL treatment samples also shifted after 7 days of aerobic exposure. These results collectively demonstrate that aerobic exposure significantly alters the bacterial community composition of the silage.

The bacterial composition of Sudan grass silage is presented in Fig. 4. At the phylum level, Firmicutes, Proteobacteria, and Bacteroidota were clearly dominant in the silages. Compared to the fresh material, all treatments increased the relative abundance of Firmicutes after 60 days of ensiling (particularly the LCX and LCL groups), while decreasing the relative abundance of Proteobacteria (except for LP) and Bacteroidota. After aerobic exposure, the relative abundance of Firmicutes decreased, while that of Proteobacteria, Bacteroidota, and Acidobacteriota increased. This shift is primarily because the low-pH environment favors the growth of bacteria within Firmicutes while inhibiting those in Proteobacteria (62). Therefore, the low pH conditions after ensiling were suitable for the growth of Firmicutes species. The subsequent rise in pH during aerobic exposure led to a reduction in the relative abundance of Firmicutes.

*Proteus*, *Bacteroides*, *Akkermansia*, and *Pantoea* were the dominant genera before ensiling, while *Lactiplantibacillus*, *Lentilactobacillus*, and *Lactococcus* became the predominant bacterial genera after 60 days of ensiling. Compared to CK, the LP and LCX treatments increased the relative abundance of *Lactiplantibacillus* (by 23.1% and 50.7%, respectively), and the LCL treatment increased the relative abundance of *Lentilactobacillus* (by 50.2%) (*P* < 0.05). Furthermore, the LCX and LCL treatments significantly reduced the relative abundances of *Enterobacter*, *Clostridium sensu stricto* 12, and *Pantoea*. Lactic acid bacilli (such as *Lactiplantibacillus* and *Lentilactobacillus*) are major producers of LA during ensiling and are typically crucial for pH reduction in the later stages of fermentation (63). *Enterobacter* and *Pantoea* belong to the Enterobacteriaceae family, and *Clostridium sensu stricto_*12 belongs to the Clostridiaceae family. These bacteria are considered undesirable in silage as they compete with LAB and other beneficial microbes for fermentable substrates, produce detrimental metabolites (eg., AN and BA), and raise the silage pH, leading to excessive nutrient losses. Additionally, *Clostridium sensu stricto* 12 can produce spores, adversely affecting livestock (43, 64). This result explains the higher pH and BA content observed in the CK and LP treatments (Fig. 1; Table 2). After 60 days of ensiling, a relatively high abundance of *Bacteroides* (12.9%) was detected in CK and then increased during the aerobic exposure in all treatments. *Bacteroides* can degrade protein and produce AN (65), which partly accounts for the highest AN content observed in CK (Table 2). Higher relative abundance of *Acetobacter* was detected in the LCX, which can cause aerobic spoilage under aerobic conditions by oxidizing LA and AA (66), which may lead to poor aerobic stability.

**Fig 5:**
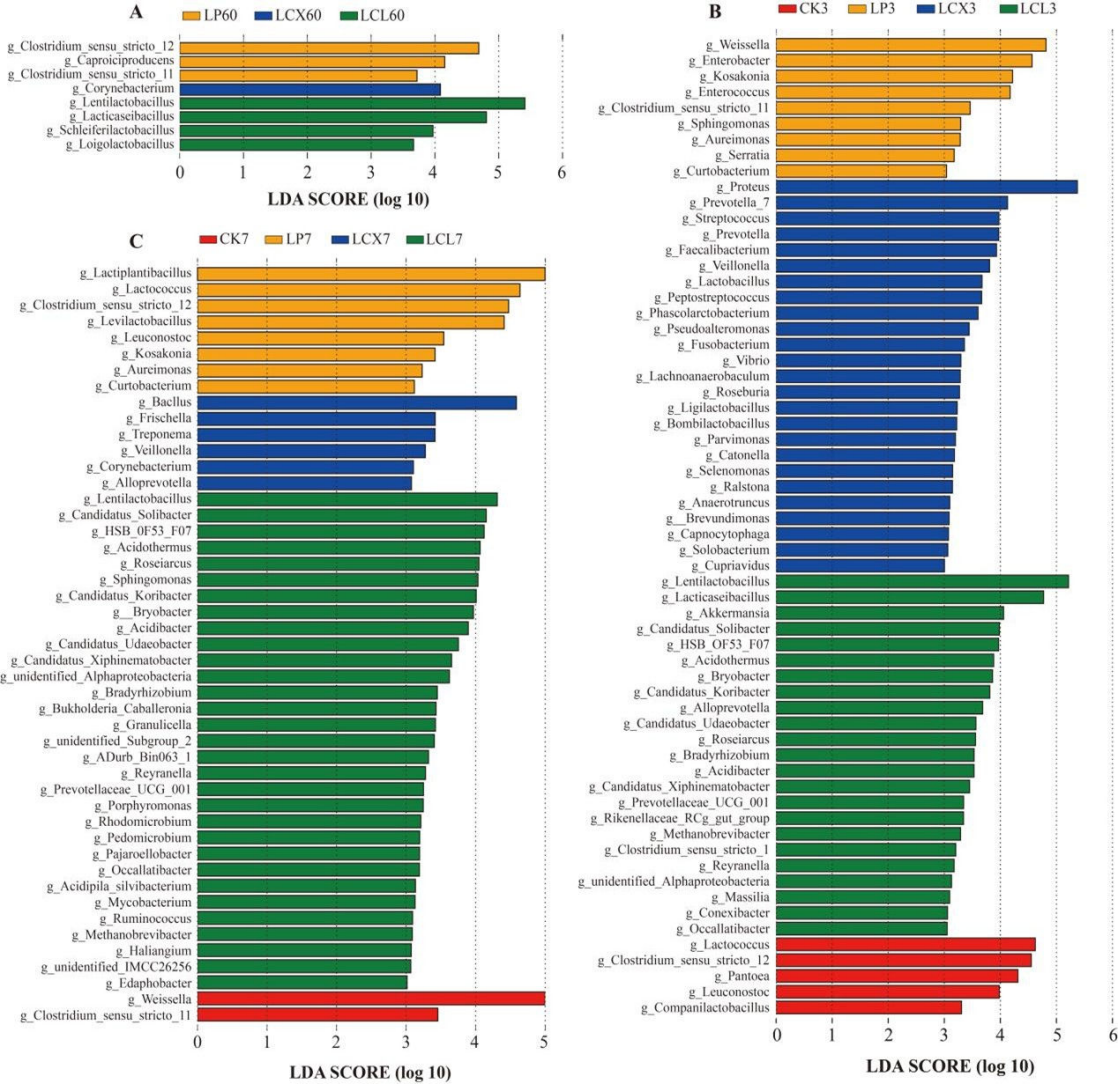
LEfSe analysis to compare the bacterial changes on 60 days and during aerobic exposure of Sudan grass silage. (**A**) Sixty days of ensiling; (**B**) aerobic exposure of 3 days; and (**C**) aerobic exposure of 7 days. LP, *L. plantarum*; LCX, *L. plantarum* + cellulase + xylanase; LCL, *L. plantarum* + cellulase + laccase.

Interestingly, the LCL treatment, which added *L. plantarum*, showed a high relative abundance of *Lentilactobacillus* and *Lacticaseibacillus* (Fig. 4B and 5), a finding consistent with results reported by Bai et al. (67). This may be attributed to the addition of laccase, which degraded lignin and produced a variety of low molecular weight aromatics such as vanillic acid, syringaldehyde, ferulic acid, and p-coumaric acid (68, 69). These compounds are either metabolized by heterotypic lactic acid bacteria or act as signaling molecules to influence their growth, thereby creating a microenvironment favorable for the growth of heterofermentative LAB and ultimately promoting their proliferation (9, 70). *Lentilactobacillus* and *Lacticaseibacillus* are heterofermentative LAB capable of converting LA to AA in the later stages of ensiling, thereby improving the aerobic stability of silage. Moreover, *Lacticaseibacillus* has a broad antibacterial spectrum, inhibiting various spoilage and pathogenic bacteria, including *Escherichia coli* (10, 47). This explains why the LCL treatment exhibited higher AA content, lower relative abundance of undesirable bacteria and fungi, and superior aerobic stability. Lignocellulose contains various phenolic compounds, and its hydrolysis can generate microbial inhibitors (such as furan derivatives and phenolic compounds) that affect microbial growth. Specifically, furan compounds can inhibit homofermentative lactobacilli while promoting the growth of heterofermentative lactobacilli (70). In contrast, cellulase and xylanase do not produce those microbial inhibitors in hydrolysates (71). Previous studies have reported a higher relative abundance of *L. buchneri* in treatments involving FAE-producing *L. plantarum*, xylanase, and laccase (9). Therefore, we speculate that laccase, possibly in synergy with other additives, may promote the production of these microbial inhibitors, thereby favoring the growth of heterofermentative LAB, such as *Lentilactobacillus* and *Lacticaseibacillus,* while suppressing the bacterial growth of *Lactiplantibacillus*.

The relatively high abundances of *Lactococcus*, *Weissella*, and *Enterococcus* were detected in the CK and LP treatments (Fig. 4B and 5). This is likely attributable to the higher pH values in these treatments, as these LAB genera primarily play important roles in initiating silage fermentation during the early stages but are relatively sensitive to low pH and are typically replaced by more acid-tolerant lactobacilli in the later fermentation phases (45). As aerobic exposure progressed, the relative abundance of lactobacilli in the LCX and LCL treatments decreased to their lowest levels by days 3 and 7, respectively. This trend correlated with the observed changes in pH and LA content and may be due to the growth of yeasts (Fig. 8), whose metabolic production of organic acids could inhibit LAB growth, and the lack of fermentable substrates for LAB after aerobic exposure (72). LEfSe analysis (Fig. 5) revealed that after 7 days of aerobic exposure, *Lentilactobacillus* remained the dominant genus in the LCL treatment, whereas *Clostridium sensu stricto_*12 became a dominant genus in both CK and LP. The presence of *Clostridium sensu stricto_*12 likely explains the higher BA content observed in CK and LP at day 7 of aerobic exposure. These bacterial community results indicate that the combined application of bacteria and enzymes better optimized the bacterial community structure of silages, leading to improved fermentation quality. Furthermore, the bacterial community in the LCL treatment demonstrated greater stability following aerobic exposure.

**Fig 6:**
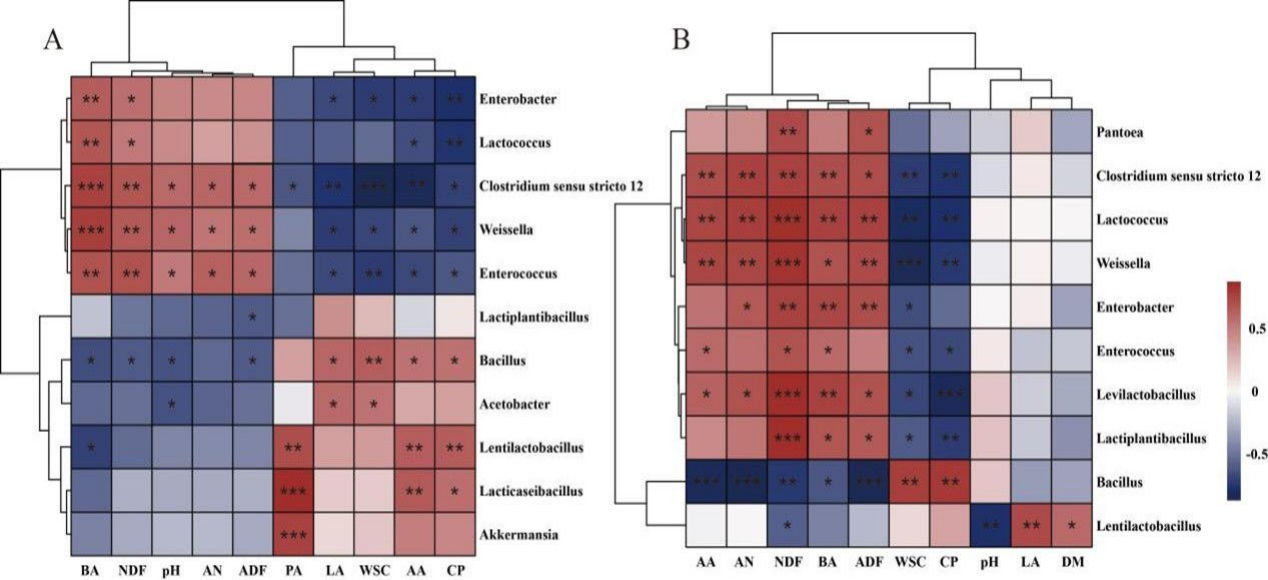
Spearman correlation heatmap based on bacterial genera and fermentation quality. (**A**) Sixty days of ensiling; (**B**) aerobic exposure of 7 days. LA, lactic acid; AA, acetic acid; PA, propionic acid; BA, butyric acid; DM, dry matter; WSC, water-soluble carbohydrates; AN, ammonia nitrogen; CP, crude protein; NDF, neutral detergent fiber; ADF, acid detergent fiber. *, *P* < 0.05; **, *P* < 0.01; ***, *P* < 0.001.

The Spearman correlation heatmap between silage parameters and the bacterial community is shown in Fig. 6. After 60 days of ensiling, *Clostridium sensu stricto_*12 showed a significantly negative correlation with LA, CP, and WSC, and a positive correlation with pH, AN, and BA (*P* < 0.05). This indicates that the bacteria likely contribute to increased pH, producing AN and BA by utilizing nutrients. Under rainy and wet conditions, adding only homofermentative FAE-producing *L. plantarum* in Sudan grass silage is insufficient to outcompete naturally occurring *Clostridium sensu stricto_*12 in the early stage. In contrast, the LCX and LCL treatments more effectively suppress *Clostridium sensu stricto_*12 by rapidly releasing WSC and accelerating acidification (73, 74). *Weissella*, *Enterococcus*, and *Enterobacter* were negatively correlated with WSC and positively correlated with pH (*P* < 0.05), suggesting that these genera may be primarily responsible for WSC depletion and an increase in pH in the silage. In general, *Weissella* and *Enterococcus* are predominantly active in the initial ensiling phase and are later replaced by acid-tolerant lactobacilli (45). *Lactiplantibacillus* and *Bacillus* exhibited a negative correlation with ADF (*P* < 0.05), which could be attributed to the ability of *Lactiplantibacillus* to produce feruloyl esterase and *Bacillus* to secrete cellulase. This also explains the lowest fiber content observed in the LCX treatment. *Lentilactobacillus* and *Lacticaseibacillus* were positively correlated with AA and PA (*P* <0.01), indicating their primary role in the production of AA and PA. After 7 days of aerobic exposure, *Weissella*, *Enterococcus*, *Lactococcus*, *Enterobacter*, and *Clostridium sensu stricto_*12 were positively correlated with NDF, ADF, and BA, and negatively correlated with WSC and CP (*P* <0.05). The elevated pH after 7 days of aerobic exposure created favorable conditions for their growth, leading to the consumption of WSC and CP and a resultant increase in NDF, ADF, and BA. *Lentilactobacillus* was positively correlated with LA and negatively correlated with pH (*P* < 0.001), which partly explains the superior aerobic stability of the LCL treatment.

### Fungal community in Sudan grass silage

**Fig 7:**
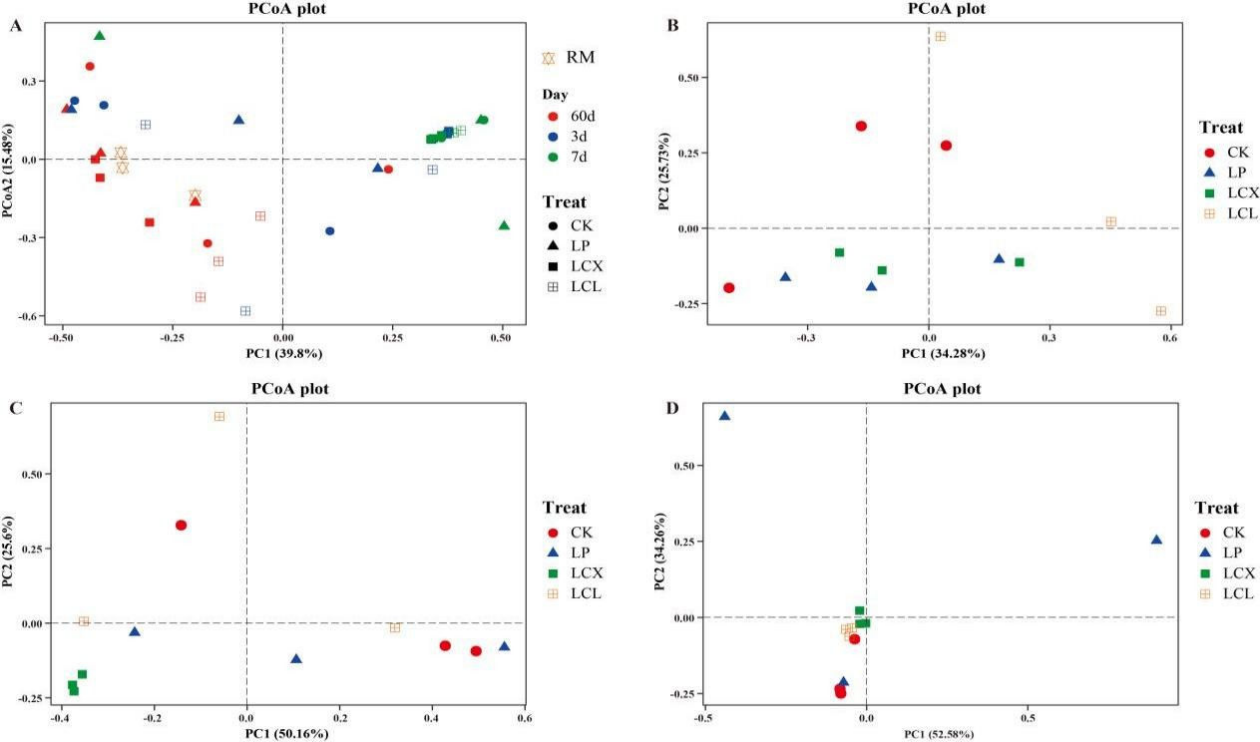
Principal coordinate analysis of fungal communities. (**A**) All time periods; (**B**) 60 days of ensiling; (**C**) aerobic exposure of 3 days; (**D**) aerobic exposure of 7 days. LP, *L. plantarum*; LCX, *L. plantarum* + cellulase + xylanase; LCL, *L. plantarum* + cellulase + laccase.

**Fig 8:**
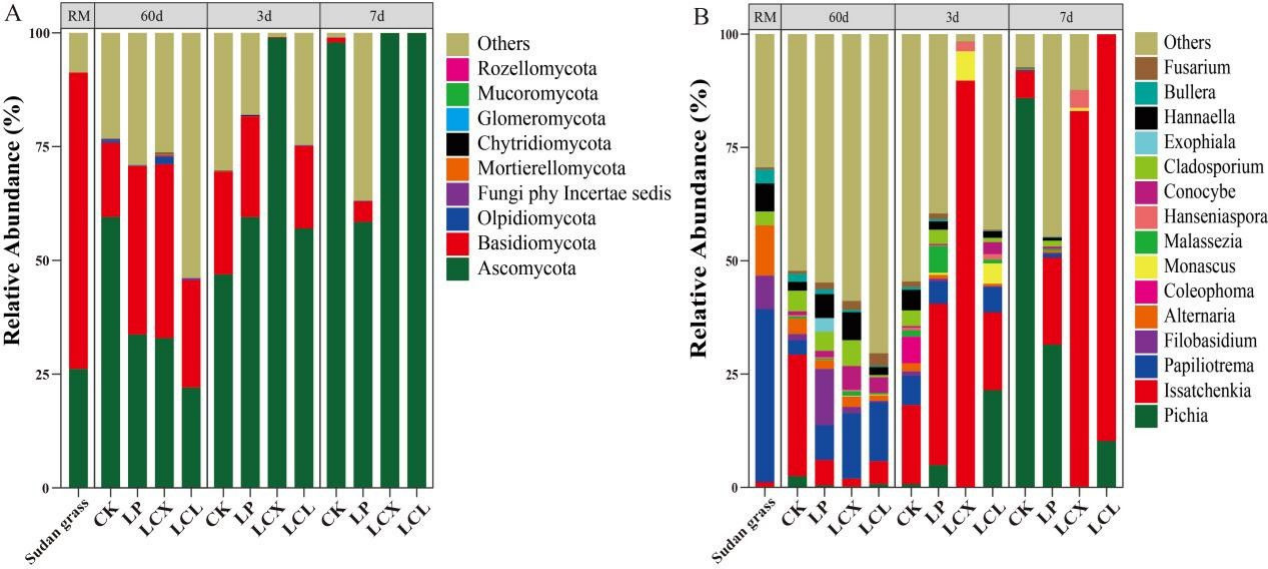
Composition of fungal communities on 60 days ensiling and during aerobic exposure of Sudan grass. (**A**) Phylum level; (**B**) genus level. LP, *L. plantarum*; LCX, *L. plantarum* + cellulase + xylanase; LCL, *L. plantarum* + cellulase + laccase.

The fungal alpha diversity of Sudan grass silage is presented in Table 3. While most studies have focused on toxin-producing fungi in silage (61), information regarding its epiphytic fungal community remains limited. After 60 days of fermentation, the LP treatments (LP, LCX, and LCL) showed no significant impact on fungal richness or diversity compared to the CK group, a finding consistent with the results reported by Bai et al. (28). After 7 days of aerobic exposure, fungal richness and diversity decreased in all treatment groups. This observation aligns with the study by Zhang et al. (75) and may be attributed to the dominance of genera such as *Pichia* or *Issatchenkia* in the later stages of aerobic exposure (Fig. 8). The principal coordinate analysis of fungal communities after 60 days of ensiling and during aerobic exposure is shown in Fig. 7. The sample points for both the fresh material and the ensiled samples were primarily clustered in the first, second, and third quadrants, suggesting that anaerobic fermentation and additive application had a relatively limited influence on the fungal community structure during ensiling. The LCL treatment samples were distributed farther away from other treatments, perhaps because of their high aerobic stability, which affected their fungal community. The LCX treatment samples clustered in the third quadrant after 3 days of aerobic exposure, showing separation from other treatments, which corresponds to the spoilage observed from day 3. By day 7 of aerobic exposure, sample points from all treatments were mainly concentrated in the third quadrant, likely due to the dominance of fungi or yeast, across all groups. These results indicate that aerobic exposure significantly altered the fungal community structure of the silage.

**Fig 9:**
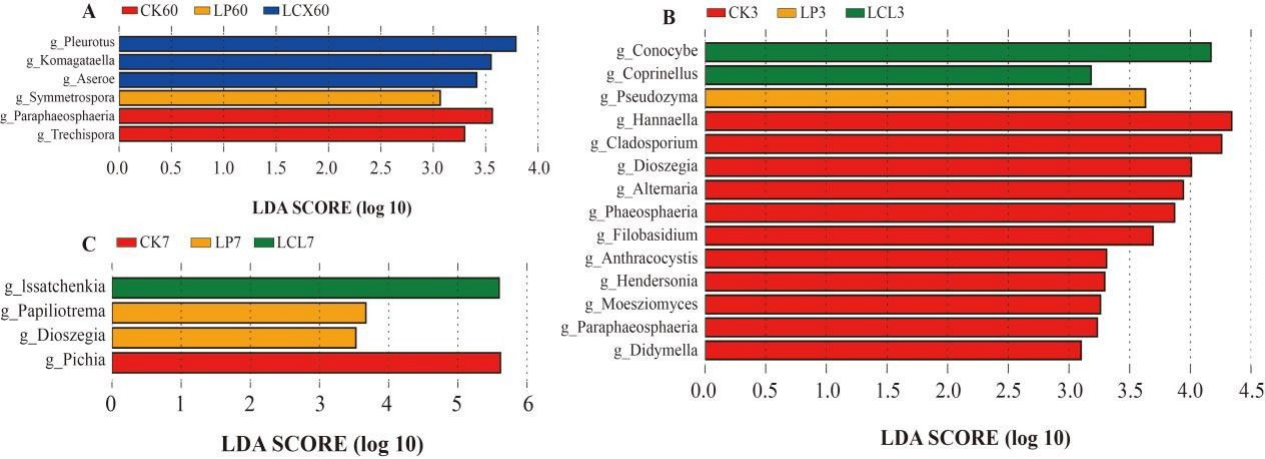
LEfSe analysis to compare the bacterial changes on 60 days and during aerobic exposure of Sudan grass silage. (**A**) Sixty days of ensiling; (**B**) aerobic exposure of 3 days; (**C**) aerobic exposure of 7 days. LP, *L. plantarum*; LCX, *L. plantarum* + cellulase + xylanase; LCL, *L. plantarum* + cellulase + laccase.

As shown in Fig. 8A, Ascomycota and Basidiomycota were clearly the dominant phyla. After 60 days of ensiling, compared to the CK, the treatments of LP, LCX, and LCL reduced the relative abundance of Ascomycota and increased the relative abundance of Basidiomycota. Liu et al. (72) similarly reported a higher relative abundance of Basidiomycota in silage treated with LAB. The relative abundance of Ascomycota increased with the duration of aerobic exposure, eventually becoming the dominant phylum, a finding consistent with the results reported by Bai et al. (28). As presented in Fig. 8B, *Papiliotrema*, *Filobasidium*, *Alternaria*, and *Hannaella* were the predominant epiphytic fungal genera in the fresh material. Their relative abundances decreased after 60 days of ensiling, likely due to the low pH and anaerobic conditions. The LCL treatment exhibited the lowest relative abundance of the undesirable fungi genera (*Pichia*, *Issatchenkia*, *Papiliotrema*, and *Filobasidium*). This may be attributed to its higher AA content, which effectively suppressed the activity of major undesirable fungi. *Issatchenkia* was the dominant genus in the CK group, whereas *Papiliotrema* was dominant in the LCX and LCL treatments. Strains within *Papiliotrema* can utilize various sugars (e.g., glucose, xylose, and sucrose) as carbon sources and produce extracellular enzymes such as amylase and xylanase (76), which may have collectively contributed to the increased WSC content. Compared to other treatments, the LCL group showed the lowest relative abundances of *Alternaria* and *Cladosporium* (Fig. 8B and 9). These genera, known producers of mycotoxins, are ubiquitous molds (77). Yeasts are widely recognized to play a major role in the aerobic spoilage of silage. They can oxidize WSC and LA, leading to spoilage and nutrient losses (78).

In this experiment, the relative abundance of yeast genera (primarily *Pichia* and *Issatchenkia*) increased after aerobic exposure. In the LCX treatment, the relative abundance of *Issatchenkia* reached its peak (89.6%) on day 3 of aerobic exposure, consistent with its poor aerobic stability. Yeasts associated with aerobic spoilage of silage can be categorized into two groups: (i) those with strong sugar fermentation capability but poor LA utilization ability (e.g., *Saccharomyces*), and (ii) lactate-assimilating yeasts, which are efficient at utilizing LA (including *Pichia* and *Issatchenkia*) (79). Interestingly, after aerobic exposure, *Pichia* became the dominant genus in the CK and LP treatments, whereas *Issatchenkia* dominated in the LCX and LCL treatments (Fig. 8B and 9C). Given the higher BA content in CK and LP and the higher LA content in LCX and LCL, we speculate that *Issatchenkia* possesses stronger competitive ability in the presence of abundant LA substrate, outcompeting other yeasts, while *Pichia* exhibits greater tolerance to BA.

**Fig 10:**
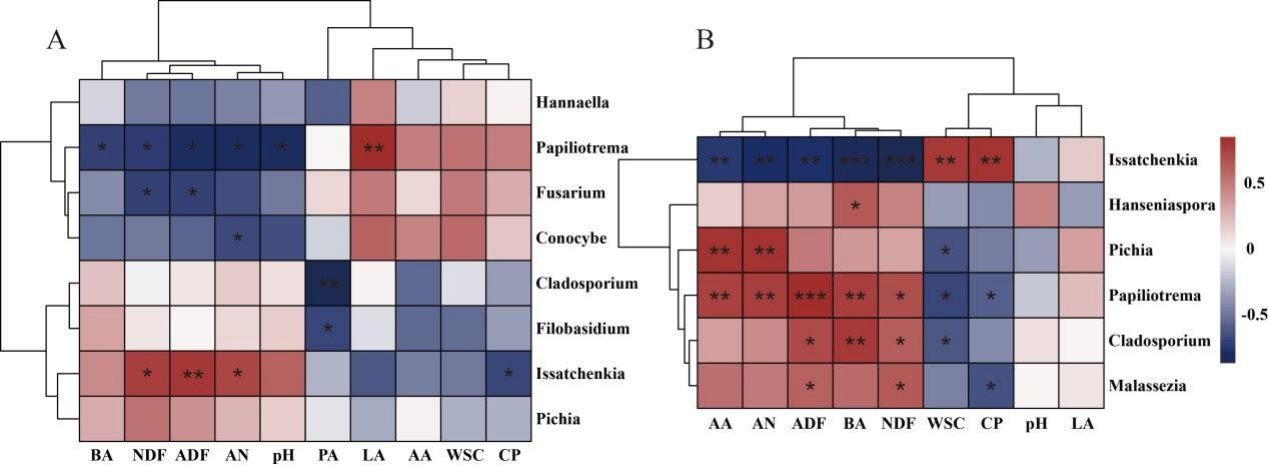
Spearman correlation heatmap based on fungal genera and fermentation quality. (**A**) Sixty days of ensiling; (**B**) aerobic exposure of 7 days. LA, lactic acid; AA, acetic acid; PA, propionic acid; BA, butyric acid; WSC, water-soluble carbohydrates; AN, ammonia nitrogen; CP, crude protein; NDF, neutral detergent fiber; ADF, acid detergent fiber. *, *P* < 0.05; **, *P* < 0.01; ***, *P* < 0.001.

The Spearman correlation heatmap is shown in Fig. 10. After ensiling for 60 days, *Issatchenkia* exhibited a negative correlation with CP and a positive correlation with AN *(P* < 0.05). Therefore, we speculate that *Issatchenkia* may utilize CP, leading to an increase in AN. After 7 days of aerobic exposure, *Issatchenkia* also showed a positive correlation with WSC and CP (*P* < 0.01), whereas *Pichia*, *Papiliotrem*a, and *Cladosporium* were negatively correlated with WSC and CP (*P* < 0.05). Moreover, *Issatchenkia* was predominantly present in the LCX and LCL treatments, while *Pichia*, *Papiliotrema*, and *Cladosporium* were mainly found in the CK and LP treatments (Fig. 8B). *Issatchenkia* is a lactate-assimilating yeast that primarily utilizes LA (78, 79). Since the LA content was very low in the CK and LP treatments, *Pichia*, *Papiliotrema*, and *Cladosporium* in these groups likely consumed WSC and CP, resulting in the observed increase in AN.

### Conclusion

Rain during harvest compromised silage quality in both CK and FAE-producing *L. plantarum* groups. In contrast, the LCX treatment significantly improved fermentation quality, evidenced by lower pH, higher LA and WSC contents, enhanced degradation of cellulose and hemicellulose, and the relative abundance of *Lactiplantibacillus*, but it exhibited the poorest aerobic stability. The LCL treatment reduced BA concentration, lignin contents, and diversity of fungal communities, while increasing AA and CP contents and the relative abundance of *Lentilactobacillus*, which contributed to the best aerobic stability exceeding 168 h. After 7 days of aerobic exposure, the LCL group showed no significant decline in nutritional composition and fermentation characteristics. Overall, adding FAE-producing *L. plantarum* with lignocellulolytic enzymes is recommended for rain-affected Sudan grass. For optimal silage quality, use LCX; for a balance of quality, stability, and lignin reduction, LCL is advised.
